# Bimodal ultrasound assessment of cerebral hemodynamics in preterm infants stratified by maternal immunotherapy: implications for early prediction of intraventricular hemorrhage

**DOI:** 10.3389/fped.2026.1854042

**Published:** 2026-07-14

**Authors:** Na Wu, Rui Cao

**Affiliations:** 1 Ultrasound Medical Center of Cangzhou Central Hospital, Cangzhou, China; 2 Department of Neonatology, Cangzhou Central Hospital, Cangzhou, China

**Keywords:** active immunotherapy, bedside ultrasound, cerebral hemodynamics, intraventricular hemorrhage, preterm infant, recurrent spontaneous abortion

## Abstract

**Objective:**

To evaluate the clinical value of bedside bimodal ultrasound in assessing early cerebral circulation in preterm infants from structural and functional perspectives, and to examine whether maternal recurrent spontaneous abortion (RSA), maternal active immunotherapy status, and ultrasound-derived markers were associated with neonatal cerebral hemodynamic trajectories and clinically significant intraventricular hemorrhage (IVH).

**Methods:**

In this single-center prospective cohort study, 120 preterm infants born to mothers with RSA were enrolled and stratified by maternal active immunotherapy status. Bedside bimodal ultrasound, including transcranial Doppler (TCD) and optic nerve sheath diameter (ONSD) measurement, was performed on postnatal days 1, 3, 5, and 7. The coefficient of variation of the middle cerebral artery pulsatility index (PI-CV) was calculated. Generalized estimating equations (GEE) were used for longitudinal analyses, and multivariable logistic regression was used to evaluate associations with clinically significant IVH (Papile grade ≥II). Because the dataset was a limited tabular cohort, no random training/validation/test split and no class-balancing procedure were used; internal validation was performed using bootstrap resampling. Model performance was assessed using discrimination, calibration, clinical-utility, and likelihood-based model-quality metrics.

**Results:**

PI-CV and ONSD decreased during the first postnatal week in both groups. GEE analysis using an AR(1) working correlation structure showed significant time effects and group × time interactions for PI-CV and ONSD (all *P* < 0.001), with a faster decline in the active immunotherapy group. ONSDmax and PI-CV at 24 h remained associated with clinically significant IVH after clinical adjustment. The bimodal model had an AUC of 0.759 (95% CI, 0.674–0.844), the clinical-ultrasound model had an AUC of 0.799 (95% CI, 0.721–0.877), and the expanded explanatory model had an AUC of 0.817 (95% CI, 0.743–0.892). For the expanded model, the likelihood-ratio chi-square was 40.915, AIC was 139.301, BIC was 161.601, McFadden pseudo-R2 was 0.249, and the apparent Brier score was 0.174. Bootstrap internal validation using 1,000 resamples yielded optimism-corrected AUC/Brier scores of 0.762/0.190 for the clinical-ultrasound model and 0.774/0.203 for the expanded explanatory model. These results support exploratory risk stratification rather than definitive clinical deployment.

**Conclusion:**

Bedside bimodal ultrasound is a feasible noninvasive approach for dynamic assessment of early cerebral hemodynamics in preterm infants. The combination of ONSDmax and PI-CV may improve exploratory early IVH risk stratification. Maternal immunotherapy status was associated with distinct longitudinal cerebral hemodynamic and ONSD trajectories, reflected by faster modeled declines in PI-CV and ONSD during the first postnatal week. However, given the observational design and associational regression models, these findings do not establish a direct protective effect of maternal immunotherapy on IVH risk. External validation and longer-term outcome studies are required before clinical implementation.

## 1 Introduction

Intraventricular hemorrhage (IVH) is one of the most serious complications in preterm infants and is strongly associated with mortality and long-term neurodevelopmental impairment. Its pathogenesis has traditionally been attributed to postnatal cerebral hemodynamic instability and the fragility of the germinal matrix vasculature (1–3). However, increasing evidence suggests that prenatal factors may also play a critical role in shaping neonatal cerebrovascular development and regulatory capacity (4, 5).

Recurrent spontaneous abortion (RSA) is commonly associated with maternal immune dysregulation and chronic inflammatory activation (6–11). These conditions may adversely affect placental perfusion and fetal vascular endothelial development, thereby influencing fetal neurodevelopment. Previous studies have suggested that maternal immune imbalance may increase the vulnerability of the neonatal brain to injury (12, 13), but its specific impact on early postnatal cerebral hemodynamics remains insufficiently understood.

These prenatal considerations intersect with postnatal vulnerability, as early fluid status and cerebral blood-flow modeling have been associated with severe IVH or IVH-risk assessment in extremely preterm infants (14, 15). Meanwhile, immunomodulatory interventions for recurrent pregnancy loss have been increasingly evaluated, but their effects and safety remain heterogeneous across study designs and patient subgroups (16–19).

Bedside ultrasound provides a practical tool for evaluating neonatal cerebral circulation. Transcranial Doppler (TCD) enables the assessment of cerebral blood flow dynamics (20, 21), while optic nerve sheath diameter (ONSD) has been proposed as a noninvasive surrogate marker of intracranial pressure (22, 23). However, a single parameter may not fully capture the complexity of cerebral circulation. A combined assessment integrating functional and structural parameters may improve early risk stratification (24, 25).

Therefore, this study aimed to dynamically evaluate early postnatal cerebral hemodynamic changes in preterm infants born to mothers with RSA using a bimodal ultrasound approach, and to investigate the association between these parameters and the risk of clinically significant IVH.

## 2 Methods

### 2.1 Study design and participants

This was a single-center prospective cohort study conducted in the neonatal intensive care unit (NICU) of Cangzhou Central Hospital between 26 November 2025 and 10 April 2026. The cohort database was locked on 10 April 2026, which preceded the manuscript's initial journal receipt date of 12 April 2026; therefore, no post-initial-submission participants or outcome data were added to the analytic cohort. All enrolled infants and all data included in the final analysis were covered by the approved protocol (Approval No. 2025-403-02). The study was approved by the Ethics Committee of Cangzhou Central Hospital, and written informed consent was obtained from the parents or legal guardians of all participating preterm infants. The study complied with the Declaration of Helsinki, Good Clinical Practice principles, and relevant national ethical regulations.

Inclusion criteria were:

(1) preterm infants with gestational age <37 weeks;
(2) maternal history of recurrent spontaneous abortion (RSA);
(3) admission to the NICU within 24 h after birth with completion of initial ultrasound assessment.

Exclusion criteria were:

(1) major congenital malformations or chromosomal abnormalities;
(2) significant congenital heart disease affecting hemodynamics;
(3) severe perinatal asphyxia requiring resuscitation for more than 10 min;
(4) incomplete clinical data or loss to follow-up.

Infants were stratified into an active immunotherapy group and a control group according to maternal treatment status before pregnancy.

### 2.2 Ultrasound assessment

Bedside bimodal ultrasound was performed according to standardized neonatal neurosonography and transcranial Doppler practice principles. All examinations were conducted by trained sonographers who were blinded to the final IVH classification. Device presets, depth, gain, sample volume, and insonation windows were kept constant whenever clinically feasible.

**Figure 1:**
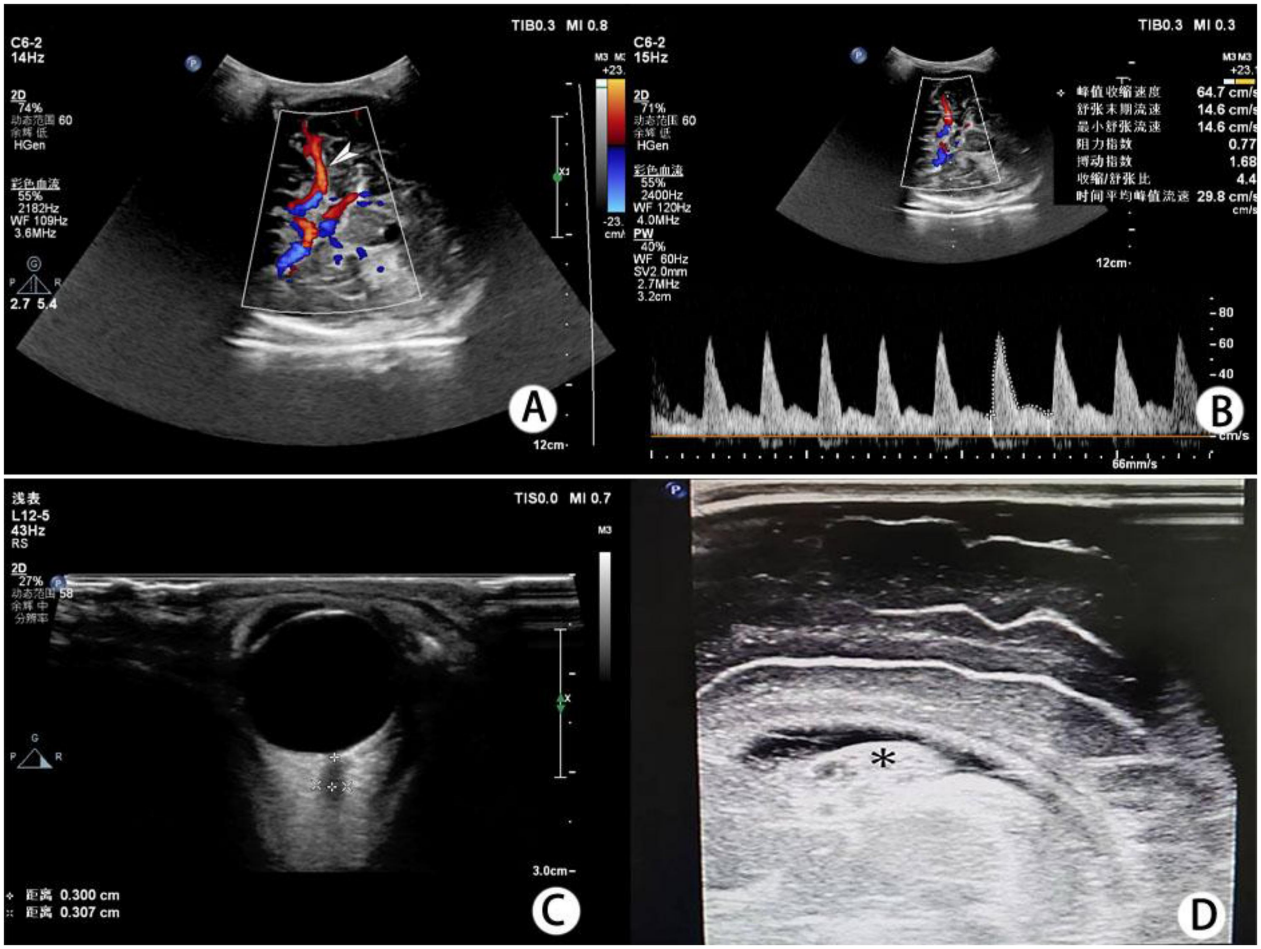
Bimodal ultrasound assessment and representative imaging findings. **(A)** Transfontanelle color Doppler ultrasound localization. The arrow indicates the M1 segment of the middle cerebral artery (MCA), which serves as the target vessel for functional assessment of cerebral hemodynamics. **(B)** Pulsed-wave Doppler spectrum of the middle cerebral artery. A stable cardiac cycle is demonstrated, allowing calculation of the pulsatility index (PI) and its coefficient of variation (PI-CV), which are used to quantify the resilience of cerebral autoregulation. **(C)** Measurement of optic nerve sheath diameter (ONSD). According to the CLOSED protocol, the diameter was measured 3 mm posterior to the globe (calipers shown), serving as a noninvasive surrogate marker of intracranial pressure. **(D)** Clinically significant IVH outcome phenotype. The neonatal sagittal section ultrasound showed grade II intraventricular hemorrhage, hyperechoic mass in the ventricle (indicated by asterisk) and normal ventricular size.

TCD was performed through the anterior fontanelle to obtain middle cerebral artery flow signals. For each examination, stable Doppler spectra from at least five consecutive cardiac cycles were recorded. The pulsatility index (PI) was calculated for each accepted cycle, and PI-CV was defined as the standard deviation divided by the mean PI multiplied by 100%, thereby quantifying short-term variability of cerebral vascular pulsatility (Figure 1 A, B).

ONSD was measured using a transorbital approach in accordance with the CLOSED protocol and recent ONSD point-of-care ultrasonography quality criteria. The diameter was measured 3 mm posterior to the globe, avoiding direct globe pressure and excessive acoustic exposure. Bilateral measurements were repeated three times when image quality allowed; the mean bilateral value and maximum value (ONSDmax) were recorded (Figure 1C).

Ultrasound examinations were performed on postnatal days 1, 3, 5, and 7. For the IVH prediction model, ONSDmax was strictly defined as the maximum bilateral value obtained within the first 24 h after birth (postnatal day 1), rather than the maximum value selected across the entire first postnatal week, to preserve its interpretation as an early bedside predictor.

#### 2.2.1 Outcome adjudication and expert panel

The expert panel supporting ultrasound image review, clinical annotation, and IVH outcome adjudication consisted of five specialists: one attending neonatologist from the NICU with 7 years of clinical experience, one attending sonographer with 25 years of ultrasound experience, one chief sonographer with 26 years of ultrasound experience, and two associate chief sonographers with 15 years of ultrasound experience each. The mean professional experience of the panel was 17.6 ± 7.9 years. This information is reported to characterize the clinical expertise underlying image annotation and outcome review.

### 2.3 Variables and definitions

The primary variables included PI-CV and ONSD, as well as the maximum ONSD (ONSDmax).

The primary outcome was clinically significant intraventricular hemorrhage (IVH), defined as Papile grade ≥II (Figure 1D).

Covariates considered for longitudinal and prediction analyses included gestational age, birth weight, mechanical ventilation, PaCO2 or PaCO2 variability, infection status, and maternal immunotherapy status. Variables retained in each model are reported explicitly below.

### 2.4 Sample size, data partitioning, and class distribution

The analytic cohort consisted of 120 infants, including 52 infants with clinically significant IVH events (43.3%) and 68 without events (56.7%). The endpoint distribution was therefore not severely imbalanced. The prediction component used structured tabular variables derived from bedside ultrasound and clinical data rather than an image-based machine-learning classifier. Given the limited sample size and event count, the cohort was not randomly divided into separate training, validation, and test sets, because such partitioning would have reduced the effective number of events for model estimation and produced unstable estimates. Instead, the full cohort was used for model fitting, and internal validation was performed using 1,000 bootstrap resamples to estimate optimism-corrected discrimination and calibration-related prediction error, reported as optimism-corrected AUC and Brier score. No undersampling, oversampling, SMOTE, class weighting, or other class-balancing strategy was applied. The prediction analysis should therefore be interpreted as exploratory internal model development and validation, not as a ready-to-use clinical decision rule.

### 2.5 Statistical analysis

Statistical analyses were performed using SPSS 29.0 and R/Python statistical software. The observational cohort component was cross-checked against STROBE reporting principles (26), and the prediction component followed TRIPOD + AI and PROBAST + AI principles where applicable for regression- or machine-learning-based prediction models (27, 28).

Continuous variables were expressed as mean ± standard deviation or median (interquartile range), depending on distribution. Normality was assessed using the Shapiro–Wilk test and inspection of Q–Q plots. Normally distributed continuous variables were compared using Student's *t*-test, non-normally distributed continuous variables and ordinal variables such as Apgar scores were compared using the Mann–Whitney *U*-test, and categorical variables were compared using the *χ*^2^ test or Fisher's exact test, as appropriate. The Z statistics reported for nonparametric comparisons represent standardized Mann–Whitney *U*-test statistics and should not be interpreted as parametric *Z*-tests.

Generalized estimating equations (GEE) with robust standard errors and a first-order autoregressive [AR(1)] working correlation structure were used to analyze repeated PI-CV and ONSD measurements. Time was coded as postnatal day (D1, D3, D5, and D7). Models included group, time, group × time interaction, gestational age, birth weight, mechanical ventilation, and simultaneously measured PaCO2. A sensitivity analysis using cubic spline terms for time and mechanical ventilation × spline-time interaction was performed to assess potential nonlinearity.

For IVH prediction, candidate variables included ONSDmax, PI-CV at 24 h, gestational age, birth weight, mechanical ventilation, PaCO2 variability, and maternal immunotherapy status. Variables were retained on the basis of prespecified clinical relevance, univariable screening (*P* < 0.10), collinearity diagnostics, and the available number of events. Model 1 was defined as the bedside clinical-ultrasound model and excluded maternal immunotherapy; Model 2 was defined as an expanded explanatory model that included maternal immunotherapy as a contextual upstream exposure. No random training/validation/test split and no class-balancing procedure were applied. Model quality was reported using convergence status, log-likelihood, likelihood-ratio chi-square, AIC, BIC, McFadden, Cox–Snell and Nagelkerke pseudo-R2, Brier score, Hosmer-Lemeshow calibration test, variance inflation factors, and bootstrap optimism correction using 1,000 resamples for both Model 1 and Model 2. Optimism-corrected AUC was used as the discrimination metric, and optimism-corrected Brier score was used as the calibration-related prediction-error metric. AUCs were compared using DeLong tests, and clinical utility was evaluated using decision curve analysis.

## 3 Results

### 3.1 Baseline characteristics

A total of 120 preterm infants born to mothers with recurrent spontaneous abortion (RSA) were included in this study, with 60 infants in the active immunotherapy group and 60 in the control group.

There were no significant differences between the two groups in maternal characteristics and perinatal factors, including maternal age, incidence of clinical chorioamnionitis, and antenatal corticosteroid use (all *P* > 0.05).

Regarding neonatal baseline demographic characteristics, no significant differences were observed in gestational age, birth weight, sex distribution, or mode of delivery between the two groups (all *P* > 0.05).

In terms of early postnatal clinical status, there were no significant differences in mean arterial pressure (MAP) at admission, incidence of patent ductus arteriosus (PDA), use of mechanical ventilation, or vasoactive drugs (all *P* > 0.05). In addition, the 5 min Apgar score was comparable between the two groups (*P* = 0.094).

No significant differences were found in baseline coagulation parameters, including platelet count (PLT) and activated partial thromboplastin time (APTT) (all *P* > 0.05).

These findings indicate that the two groups were comparable at baseline, providing a reliable basis for subsequent analyses (Table 1).

**Table 1:** Baseline characteristics of preterm infants stratified by maternal immunotherapy.

Variable	Active immunotherapy (*n* = 60)	Control (*n* = 60)	Statistic	*P* value
Sex, *n* (%)			*χ*^2^ = 0.541	0.462
Male	36 (60.0)	31 (51.7)		
Female	24 (40.0)	29 (48.3)		
Maternal age, years	30 (26–33)	29 (27–31)	MWU *Z* = 0.262	0.795
Apgar score (1 min)	7 (6–7)	6 (6–7)	MWU *Z* = 1.564	0.097
Apgar score (5 min)	8 (7–8)	7 (7–8)	MWU *Z* = 1.580	0.094
Vasoactive drug use, *n* (%)	11 (18.3)	17 (28.3)	χ^2^ = 1.165	0.281
Clinical chorioamnionitis, *n* (%)	5 (8.3)	8 (13.3)	χ^2^ = 0.345	0.557
Patent ductus arteriosus, *n* (%)	22 (36.7)	24 (40.0)	χ^2^ = 0.035	0.851
Antenatal steroid use, *n* (%)	33 (55.0)	34 (56.7)	χ^2^ = 0.000	1.000
Mean arterial pressure (mmHg)	35 (33–38)	34 (32–36)	MWU *Z* = 1.044	0.296
Birth weight (g)	1,255 (1,198–1,330)	1,240 (1,165–1,325)	MWU *Z* = 0.541	0.590
Mechanical ventilation, *n* (%)	25 (41.7)	29 (48.3)	χ^2^ = 0.303	0.582
Gestational age (weeks)	30.9 ± 1.4	30.6 ± 1.6	*t* = 1.073	0.286
Mode of delivery, *n* (%)			χ^2^ = 0.000	1.000
Cesarean section	37 (61.7)	38 (63.3)		
Vaginal delivery	23 (38.3)	22 (36.7)		
Platelet count (×10^9^/L)	211 ± 27	202 ± 26	*t* = 1.885	0.062
APTT (s)	56 (54–60)	57 (54–59)	MWU *Z* = −0.276	0.784

MAP, mean arterial pressure; PLT, platelet count; APTT, activated partial thromboplastin time.

Data are presented as mean ± standard deviation, median (interquartile range), or number (percentage), as appropriate.

Comparisons between groups were performed using Student's *t*-test, Mann–Whitney *U*-test, χ^2^ test, or Fisher's exact test, as appropriate. Apgar scores were treated as ordinal variables and compared using the Mann–Whitney *U*-test.

### 3.2 Longitudinal changes in cerebral hemodynamic parameters

During serial assessments on postnatal days 1, 3, 5, and 7, both PI-CV and ONSD showed a decreasing trend in both groups.

Generalized estimating equations (GEE) analysis demonstrated a significant time effect for both PI-CV and ONSD (both *P* < 0.001), indicating a progressive decline in cerebral hemodynamic parameters over time.

A significant group effect was also observed, with lower overall levels of PI-CV and ONSD in the active immunotherapy group compared with the control group (both *P* < 0.001). In addition, a significant interaction between time and group was identified (interaction effect, *P* < 0.001), suggesting different temporal patterns between the two groups.

Further comparisons showed that the decline in PI-CV and ONSD was more pronounced in the active immunotherapy group.

**Figure 2:**
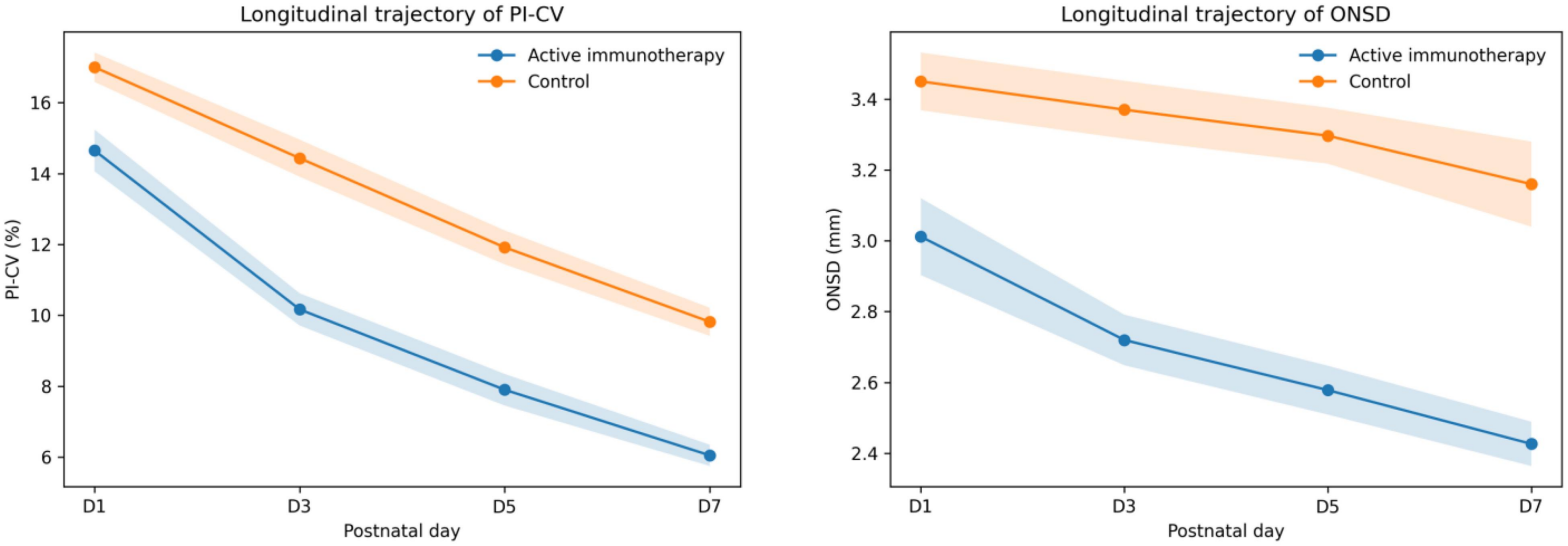
Longitudinal trajectories of cerebral hemodynamics in preterm infants. Lines show mean values and shaded areas indicate 95% confidence intervals. Postnatal time was displayed as D1, D3, D5, and D7 rather than as a continuous 120-da*y* axis. Longitudinal changes were analyzed using GEE with robust standard errors and AR(1) working correlation. Significant group × time interactions were observed for both PI-CV and ONSD (*P* < 0.001).

These findings indicate dynamic changes in cerebral hemodynamics during early postnatal life, with distinct patterns associated with maternal immunotherapy status (Table 2, Figure 2).

**Table 2:** Generalized estimating equation (GEE) analysis of longitudinal changes in PI-CV and ONSD in preterm infants.

Variable	*β*	Robust SE	Wald χ^2^	*P* value	95% CI	Interpretation
PI-CV						
Active immunotherapy group	−2.569	0.390	43.363	<0.001	−3.334 to −1.804	Lower PI-CV level
Time, per postnatal day	−1.149	0.047	589.2	<0.001	−1.242 to −1.056	Declining trajectory
Group × time	−0.225	0.057	15.468	<0.001	−0.336 to −0.113	Faster decline
Gestational age, weeks	−0.153	0.071	4.657	0.031	−0.293 to −0.014	Lower PI-CV
Birth weight, per 100 g	0.157	0.074	4.477	0.034	0.012 to 0.303	Higher PI-CV
Mechanical ventilation	−0.641	0.236	7.371	0.007	−1.103 to −0.178	Lower short-term variability
PaCO2, mmHg	0.035	0.019	3.341	0.067	−0.003 to 0.073	Borderline effect
ONSD						
Active immunotherapy group	−1.137	0.215	27.96	<0.001	−1.559 to −0.715	Lower ONSD level
Time, per postnatal day	−0.951	0.032	882.5	<0.001	−1.014 to −0.888	Declining trajectory
Group × time	−0.143	0.041	12.16	<0.001	−0.224 to −0.062	Faster decline
Gestational age, weeks	−0.082	0.061	1.80	0.180	−0.202 to 0.038	NS
Birth weight, per 100 g	0.038	0.062	0.38	0.539	−0.084 to 0.160	NS
Mechanical ventilation	−0.411	0.226	3.31	0.069	−0.854 to 0.032	Borderline effect
PaCO2, mmHg	0.008	0.011	0.53	0.467	−0.014 to 0.030	NS

GEE models were fitted with robust standard errors and a first-order autoregressive [AR(1)] working correlation structure to account for within-infant repeated measurements across D1, D3, D5, and D7. The models included group, postnatal time, group × time interaction, gestational age, birth weight, mechanical ventilation, and simultaneously measured PaCO2; cubic spline sensitivity analyses were additionally performed to examine potential nonlinear time effects.

NS, not significant; PI-CV, coefficient of variation of pulsatility index; ONSD, optic nerve sheath diameter; PaCO2, partial pressure of arterial carbon dioxide.

### 3.3 Factors associated with clinically significant IVH

Multivariable logistic regression was performed with clinically significant IVH (Papile grade ≥II) as the dependent variable. Candidate variables and reasons for inclusion were reported explicitly: ONSDmax and PI-CV were prespecified ultrasound markers; gestational age, birth weight, mechanical ventilation, and PaCO2 variability were clinically relevant covariates; maternal immunotherapy status was evaluated only as a contextual upstream exposure rather than as a directly deployable bedside predictor.

In the clinical-ultrasound model excluding maternal treatment status, ONSDmax (OR = 1.201 per 0.1 mm increase, 95% CI: 1.074–1.342, *P* = 0.001), PI-CV (OR = 1.283, 95% CI: 1.046–1.575, *P* = 0.017), mechanical ventilation (OR = 2.483, 95% CI: 1.023–6.026, *P* = 0.044), and lower gestational age (OR = 0.736 per week, 95% CI: 0.549–0.988, *P* = 0.041) remained associated with clinically significant IVH after adjustment. These estimates should be interpreted as adjusted associations, not as proof of causal effects.

The expanded explanatory model including maternal immunotherapy status achieved slightly higher apparent discrimination but yielded an imprecise treatment-status coefficient after adjustment for ONSDmax and PI-CV. Given the observational design, limited sample size, and wide confidence interval, this coefficient was not interpreted as evidence of either a harmful or protective independent causal effect. Maternal immunotherapy was therefore discussed only as a contextual exposure associated with longitudinal hemodynamic trajectories.

Collinearity was low for all predictors (all variance inflation factors <2), and the expanded model converged appropriately.

These findings support the association of ONSDmax and PI-CV with clinically significant IVH, while estimates for mechanical ventilation and maternal immunotherapy require cautious interpretation because of their wide confidence intervals and the exploratory model setting (Table 3). The change in estimates after adding maternal immunotherapy to Model 2 indicates sensitivity to model specification, but this pattern does not establish mediation, suppression, or causality (Table 4).

**Table 3:** Multivariable logistic regression analysis of factors associated with clinically significant IVH.

Domain	Predictor	Model 1 OR (95% CI)	*P* value	Model 2 OR (95% CI)	*P* value
Ultrasound-derived	ONSDmax, per 0.1-mm increase	1.201 (1.074–1.342)	0.001	1.277 (1.118–1.459)	<0.001
Ultrasound-derived	PI-CV at 24 h	1.283 (1.046–1.575)	0.017	1.451 (1.129–1.865)	0.004
Clinical	Mechanical ventilation, yes vs. no	2.483 (1.023–6.026)	0.044	2.781 (1.117–6.927)	0.028
Clinical	Gestational age, per week	0.736 (0.549–0.988)	0.041	0.730 (0.544–0.980)	0.036
Clinical	Birth weight, per 100 g	0.944 (0.687–1.298)	0.723	0.914 (0.661–1.265)	0.589
Clinical	PaCO2 variability	0.983 (0.877–1.102)	0.765	0.995 (0.887–1.116)	0.929
Contextual exposure	Maternal immunotherapy, yes vs. no	—	—	3.413 (1.006–11.578)	0.049

Model 1 was the bedside clinical–ultrasound prediction model and excluded maternal immunotherapy. Model 2 was an expanded explanatory model that included maternal immunotherapy as a contextual upstream exposure. The wide confidence intervals for mechanical ventilation and maternal immunotherapy indicate limited precision; therefore, these estimates should be interpreted as exploratory associations rather than definitive effects.

IVH, intraventricular hemorrhage; ONSD, optic nerve sheath diameter; PI-CV, coefficient of variation of pulsatility index; OR, odds ratio; CI, confidence interval. The dependent variable was clinically significant IVH (Papile grade ≥II). A two-sided *P* value < 0.05 was considered statistically significant. PI-CV was measured at 24 h after birth (postnatal day 1).

**Table 4:** Logistic regression model-quality metrics and internal validation results.

Metric	Clinical–ultrasound model (Model 1)	Expanded explanatory model (Model 2)
Number of infants	120	120
Events/non-events	52/68	52/68
Candidate predictors retained	6	7
Convergence	Yes	Yes
Maximum VIF	<2	<2
Log-likelihood	−63.832	−61.651
Null log-likelihood	−82.108	−82.108
−2 log-likelihood	127.665	123.301
Likelihood-ratio χ^2^	36.551 (df = 6)	40.915 (df = 7)
Likelihood-ratio test *P* value	<0.001	<0.001
AIC	141.665	139.301
BIC	161.177	161.601
McFadden pseudo-R^2^	0.223	0.249
Cox–Snell pseudo-R^2^	0.263	0.289
Nagelkerke pseudo-R^2^	0.352	0.388
Apparent Brier score	0.181	0.174
Hosmer–Lemeshow test	χ^2^ = 6.460, df = 8, *P* = 0.596	χ^2^ = 7.939, df = 8, *P* = 0.439
Bootstrap resamples	1,000	1,000
Bootstrap optimism-corrected AUC	0.762	0.774
Bootstrap optimism-corrected Brier score	0.190	0.203

Model-quality metrics were calculated from the final fitted logistic regression models using the same variables and scaling as Table 3. The apparent Brier score was calculated as the mean squared difference between observed IVH status and model-predicted probability. Hosmer-Lemeshow calibration testing used 10 risk groups. AIC, Akaike information criterion; BIC, Bayesian information criterion; VIF, variance inflation factor; AUC, area under the receiver operating characteristic curve.

### 3.4 Model performance and validation

#### 3.4.1 Discrimination and internal validation

ROC analysis showed moderate discrimination for ONSDmax (AUC = 0.733, 95% CI: 0.644–0.822) and PI-CV (AUC = 0.714, 95% CI: 0.621–0.807). At the Youden cutoff, ONSDmax had a sensitivity of 65.4% and specificity of 72.1%; this threshold should therefore be treated as an exploratory risk-stratification point rather than a stand-alone diagnostic cutoff.

**Figure 3:**
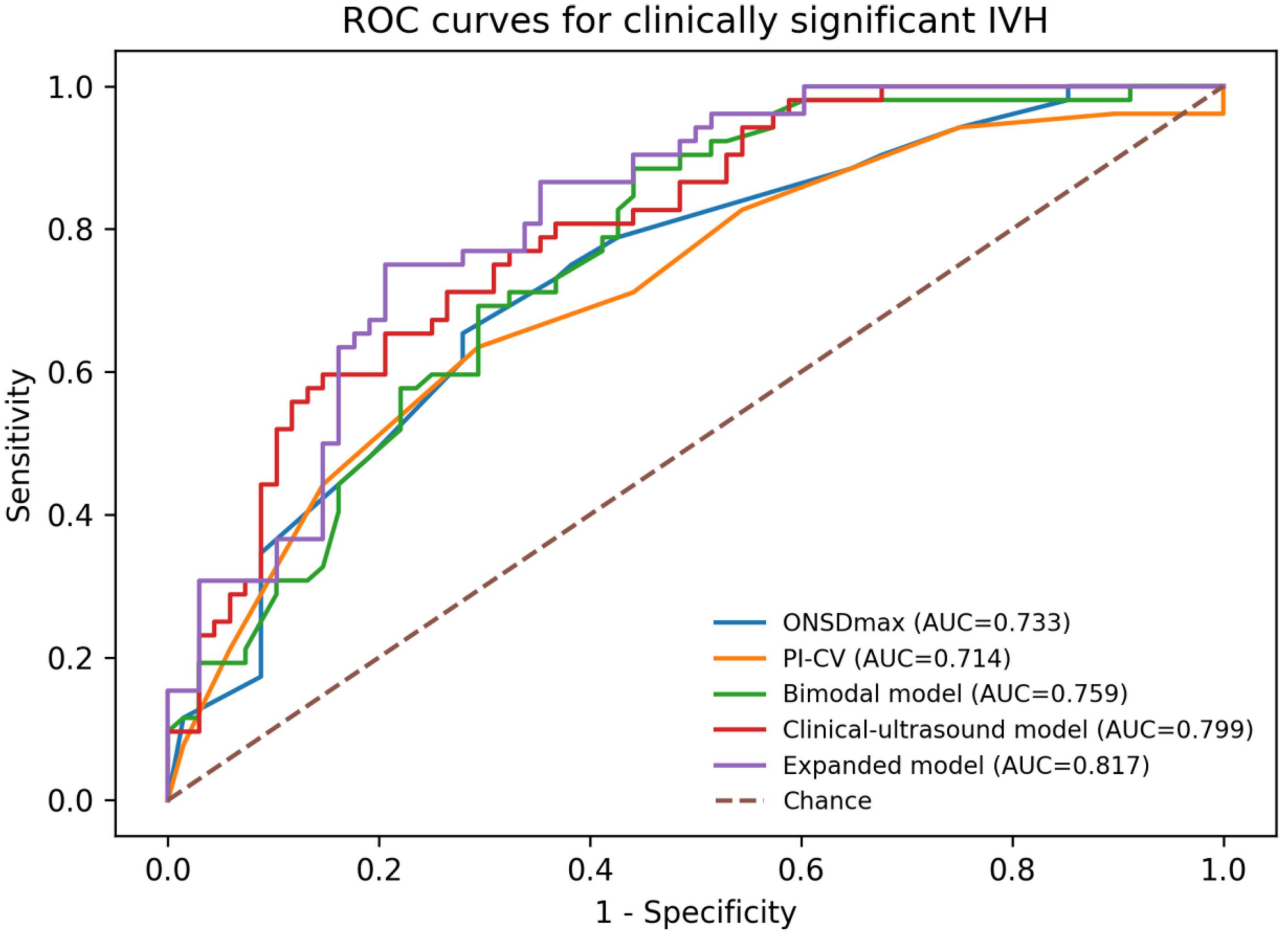
Receiver operating characteristic (ROC) curves for prediction of intraventricular hemorrhage. The ROC curve compares the bimodal ultrasound model and the expanded explanatory model. The expanded model shows higher apparent discriminative performance in this cohort. The diagonal dashed line represents the chance level.

The bimodal model combining ONSDmax and PI-CV improved apparent discrimination (AUC = 0.759, 95% CI: 0.674–0.844). The clinical-ultrasound model excluding treatment status achieved an AUC of 0.799 (95% CI: 0.721–0.877), and the expanded explanatory model (Table 5, Figure 3) achieved an AUC of 0.817 (95% CI: 0.743–0.892). In apparent DeLong comparisons, the expanded model showed higher discrimination than the bimodal model (*P* = 0.048), ONSDmax alone (*P* = 0.016), and PI-CV alone (*P* = 0.021). Bootstrap internal validation (1,000 resamples) was performed for both models. For Model 1, the optimism-corrected AUC was 0.762 and the optimism-corrected Brier score was 0.190. For Model 2, the optimism-corrected AUC was 0.774 and the optimism-corrected Brier score was 0.203. These internally validated estimates indicate modest optimism in the apparent performance and reinforce that the models should be interpreted as exploratory risk-stratification tools requiring external validation.

**Table 5:** Discriminative performance of different models for predicting clinically significant IVH.

Model	AUC	95% CI	Sensitivity	Specificity
ONSDmax	0.733	0.644–0.822	0.654	0.721
PI-CV	0.714	0.621–0.807	0.635	0.706
Bimodal model	0.759	0.674–0.844	0.885	0.559
Clinical-ultrasound model	0.799	0.721–0.877	0.596	0.853
Expanded model	0.817	0.743–0.892	0.750	0.794

IVH, intraventricular hemorrhage; AUC, area under the receiver operating characteristic curve; CI, confidence interval; ONSDmax, maximum optic nerve sheath diameter; PI-CV, coefficient of variation of pulsatility index.

Clinically significant IVH was defined as Papile grade ≥ II.

The bimodal model included ONSDmax and PI-CV measured at 24 h after birth.

The expanded model incorporated ultrasound parameters, clinical covariates, and maternal immunotherapy status.

Sensitivity and specificity were calculated at the optimal cutoff determined by the Youden index.

#### 3.4.2 Calibration

**Figure 4:**
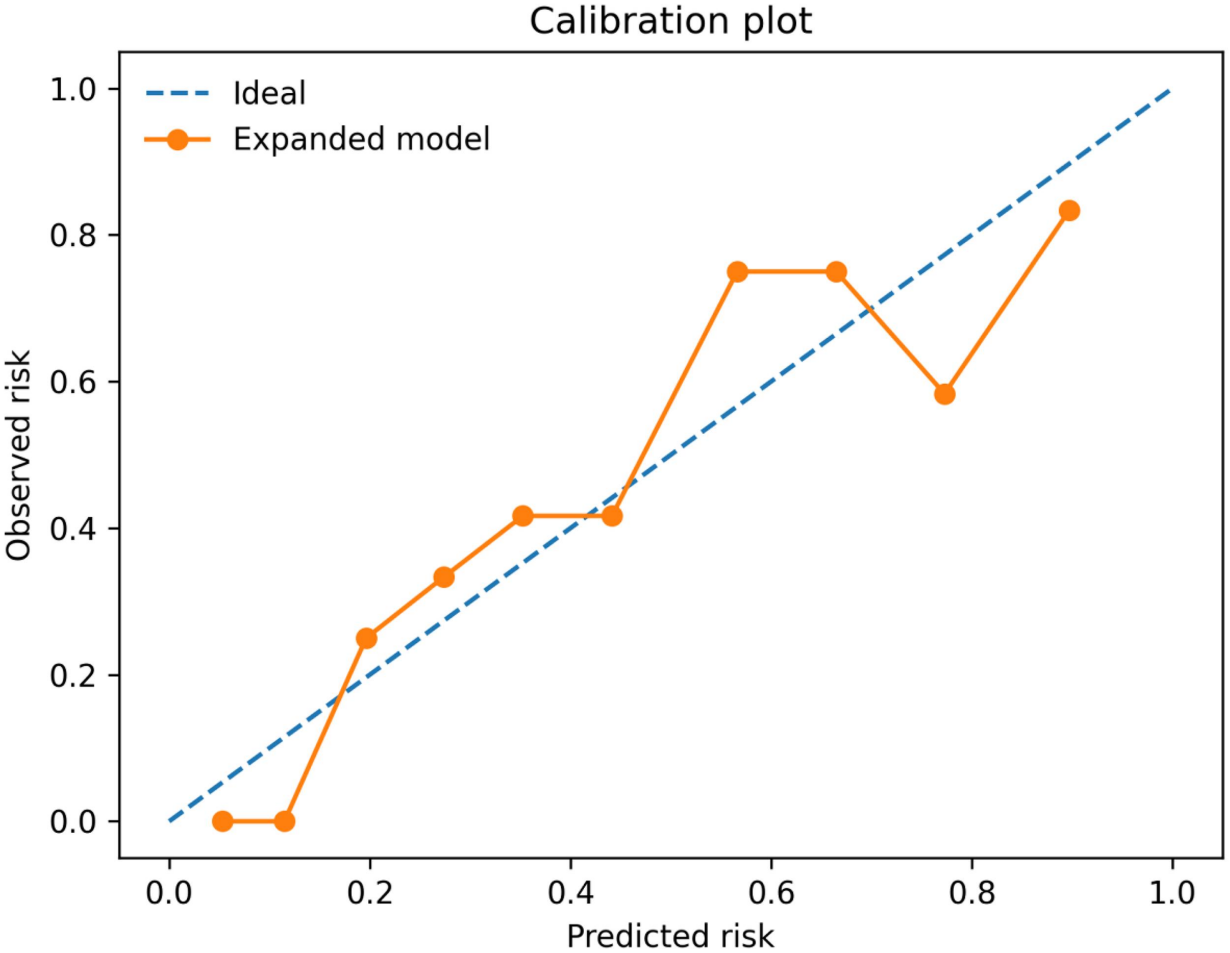
Calibration curve of the expanded explanatory model. Calibration plot comparing predicted probabilities with observed outcomes. The dashed diagonal line represents perfect calibration. The plot suggests acceptable apparent agreement between predicted and observed risks in the development cohort.

Calibration plots showed acceptable apparent agreement between predicted and observed probabilities. The Hosmer-Lemeshow test did not indicate significant lack of fit for either the clinical-ultrasound model (chi-square = 6.460, df = 8, *P* = 0.596) or the expanded explanatory model (chi-square = 7.939, df = 8, *P* = 0.439), suggesting adequate apparent calibration within this exploratory cohort (Figure 4).

#### 3.4.3 Decision curve analysis

**Figure 5:**
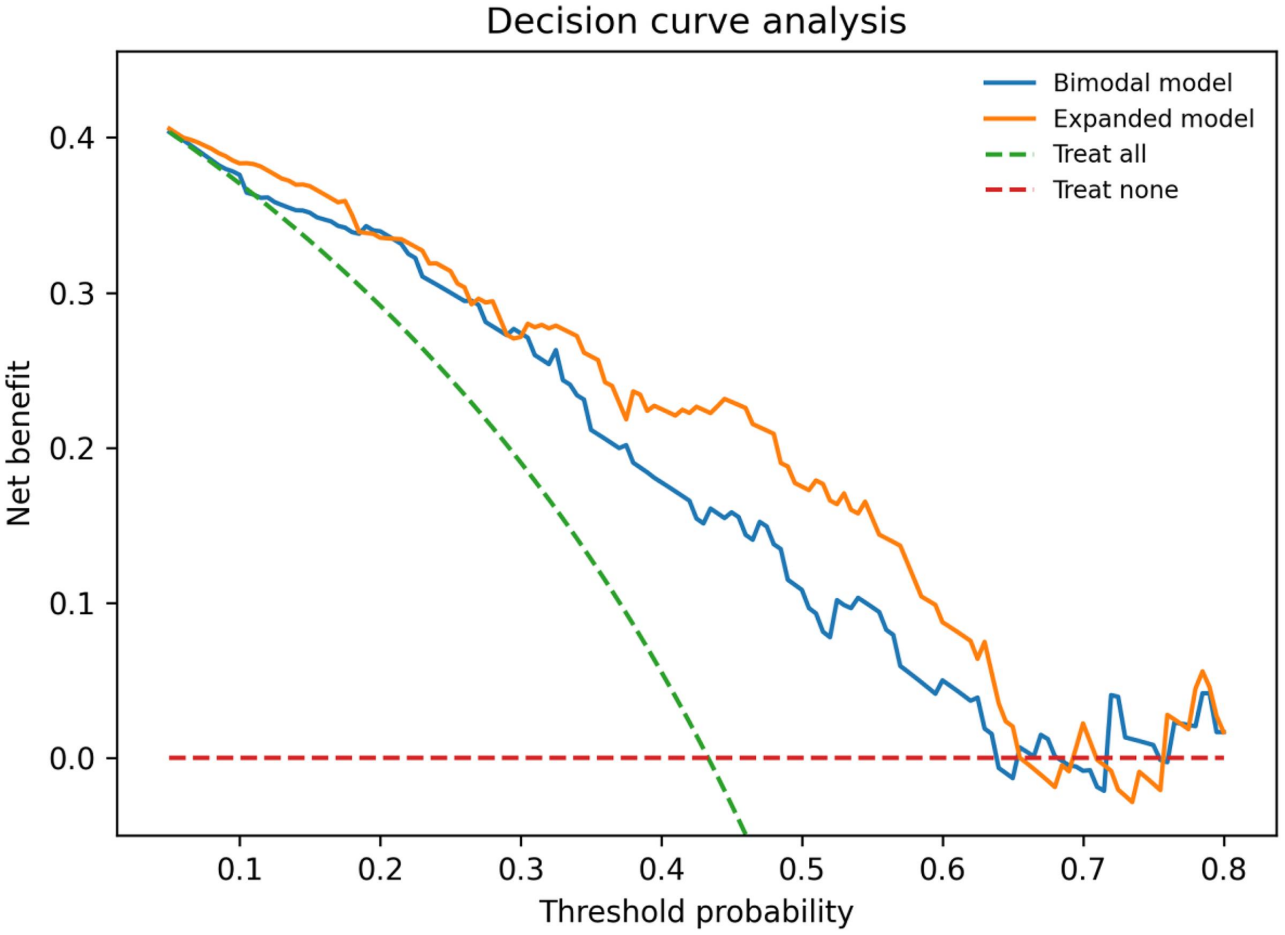
Decision curve analysis (DCA) of predictive models. Decision curve analysis showing net benefit across threshold probabilities. The bimodal and expanded explanatory models provided greater apparent clinical net benefit within clinically relevant threshold ranges. The dashed line represents the treat-all strategy, and the horizontal line represents the treat-none strategy.

Decision curve analysis (DCA) suggested that both the bimodal model and the expanded explanatory model provided higher net benefit than treat-all or treat-none strategies across a range of clinically plausible threshold probabilities (Figure 5).

Taken together, the discrimination, calibration, Brier score, and decision-curve findings indicate that ultrasound-based models incorporating ONSDmax and PI-CV provide clinically interpretable exploratory risk-stratification information. However, the modest sample size, optimism-corrected performance, and absence of external validation mean that the model should not be used for routine clinical decision-making before independent validation.

## 4 Discussion

### 4.1 Principal findings

In this prospective cohort study, we dynamically evaluated early postnatal cerebral hemodynamic changes in preterm infants born to mothers with recurrent spontaneous abortion (RSA) using a bimodal ultrasound approach. Both PI-CV and ONSD showed a decreasing trend during the first postnatal week, suggesting gradual stabilization of cerebral circulation, which is consistent with previous observations of postnatal hemodynamic adaptation in preterm infants (29, 30).

Compared with the control group, infants in the active immunotherapy group exhibited a more pronounced decline in PI-CV and ONSD, suggesting a faster pattern of early hemodynamic stabilization. In addition, ONSDmax and PI-CV measured at 24 h after birth remained associated with clinically significant IVH after adjustment, and their combination improved apparent predictive performance, consistent with prior studies linking cerebral blood flow variability and structural indicators with IVH risk (31, 32).

These findings suggest that combined structural and functional bedside ultrasound assessment may enhance early, noninvasive IVH risk stratification when interpreted together with clinical context.

### 4.2 Association between maternal immunotherapy and neonatal cerebral hemodynamics

RSA is commonly associated with maternal immune dysregulation and chronic inflammation, which may influence placental function and fetal vascular development (6, 7). Previous studies have suggested that adverse intrauterine environments may increase the susceptibility of neonates to brain injury (10, 12).

In this study, maternal active immunotherapy was associated with faster longitudinal declines in PI-CV and ONSD. In contrast, the treatment-status coefficient in the expanded IVH model was imprecise after adjustment for proximal ultrasound markers. This pattern is best interpreted as uncertainty related to model specification and residual confounding within an observational dataset, not as evidence that maternal immunotherapy directly increases or decreases IVH risk. Accordingly, we avoid describing maternal immunotherapy as an independent direct protective factor for IVH.

Because this was an observational cohort study, causality cannot be established from logistic regression alone. We did not perform formal causal modeling, Granger causality, transfer entropy, or mediation analysis; such analyses would require larger datasets, clearer temporal ordering of exposures and candidate mediators, and prespecified causal assumptions. The biological mechanisms therefore remain uncertain and may involve immune modulation, placental perfusion, fetal vascular development, and postnatal cerebral autoregulatory adaptation.

### 4.3 Clinical relevance of PI-CV and ONSD

In this study, PI-CV and ONSD were used to reflect functional and structural aspects of cerebral circulation, respectively. PI-CV represents the variability of cerebral blood flow and may reflect the stability of cerebral autoregulation, while ONSD serves as a surrogate marker of intracranial pressure, as supported by previous neurocritical care studies (22, 23).

Both parameters were associated with IVH, suggesting that functional instability and structural changes may jointly identify infants with greater vulnerability to early brain injury, consistent with current understanding of impaired cerebral autoregulation in preterm infants (29).

The combined model showed better apparent discrimination than either single marker, supporting a pragmatic multimodal approach to early bedside risk stratification rather than reliance on a single cutoff (25).

### 4.4 Interpretation of mechanical ventilation and nonlinear sensitivity findings

Mechanical ventilation showed different associations across longitudinal and binary-outcome models. In GEE analysis, ventilated infants had lower short-term PI-CV after adjustment for PaCO2, whereas mechanical ventilation was associated with higher odds of clinically significant IVH in the logistic model.

To address the possibility of nonlinearity, cubic spline terms for postnatal time and mechanical ventilation × spline-time interactions were added as sensitivity analyses. These nonlinear interaction terms were not statistically significant for PI-CV or ONSD, and the group × time findings remained directionally unchanged. The apparent discrepancy is therefore more plausibly explained by differences in analytic target: short-term physiologic stabilization under ventilatory support in the repeated-measures model vs. overall illness severity in the IVH outcome model.

Therefore, PI-CV should be interpreted cautiously in ventilated infants, and structural indicators such as ONSD should be considered alongside clinical severity rather than in isolation.

### 4.5 Clinical implications

Bimodal ultrasound is noninvasive, repeatable, and feasible in neonatal intensive care settings, and is increasingly recognized as an important bedside neuromonitoring tool (21, 22).

Measurements obtained within the first 24 h after birth may provide clinically useful information for early risk stratification. The combined use of PI-CV and ONSD may help identify infants who warrant closer neurosonographic surveillance and individualized neuroprotective attention, although the present findings do not justify treatment decisions based solely on model-predicted risk (33, 34).

The bimodal model prioritized sensitivity (0.885) at the cost of lower specificity (0.559), which may be clinically acceptable for an early screening tool where missed high-risk infants are a major concern. By contrast, the clinical-ultrasound and expanded models showed a more balanced sensitivity-specificity profile. Decision curve analysis suggested greater apparent net benefit than treat-all or treat-none strategies across clinically plausible threshold ranges. Given the event rate of 43.3% and the single-center setting, this finding should be interpreted as exploratory decision-support evidence rather than a ready-to-use clinical rule.

### 4.6 Limitations

This study has several limitations. First, it was a single-center prospective cohort study with 120 infants and 52 events; no independent training, validation, or test set was available, and bootstrap validation cannot replace external validation. Second, although the endpoint distribution was not severely imbalanced, the limited sample size produced wide confidence intervals for several predictors, particularly mechanical ventilation and maternal immunotherapy. Third, maternal immunotherapy was observational rather than randomized, leaving room for residual confounding and indication bias; no causal modeling was performed. Fourth, biological markers, placental pathology, and direct measures of intracranial pressure or cerebral autoregulation were not included, limiting mechanistic inference. Fifth, although the expert panel was characterized in terms of composition and experience (17.6 ± 7.9 years), single-center image annotation and outcome review may still limit generalizability. Finally, long-term neurodevelopmental outcomes were not assessed. These limitations are consistent with domains emphasized in PROBAST + AI for prediction-model risk-of-bias and applicability assessment (28). The need for external validation is also consistent with broader perinatal risk-prediction literature (35).

### 4.7 Future directions

Future studies should include multicenter cohorts, prespecified external validation, clearly separated training/validation/test datasets when sample size permits, biological and placental markers, standardized ultrasound acquisition audits, transparent expert-annotation reporting, and long-term neurodevelopmental follow-up. Larger datasets would also permit prespecified causal or mediation analyses to evaluate whether maternal immune modulation, cerebral hemodynamic markers, and IVH risk are connected through biologically plausible pathways.

## 5 Conclusion

This study shows that PI-CV and ONSD change dynamically during the first postnatal week and are associated with clinically significant IVH in preterm infants born to mothers with RSA. A bimodal ultrasound approach combining structural and functional information improved apparent early risk stratification compared with single parameters.

Maternal immunotherapy status was associated with distinct longitudinal cerebral hemodynamic and ONSD trajectories, reflected by faster modeled declines in PI-CV and ONSD during the first postnatal week. However, given the observational design and associational regression models, these findings do not establish a direct protective effect of maternal immunotherapy on IVH risk. The findings should therefore be interpreted as hypothesis-generating.

The proposed ultrasound-based model may serve as a practical exploratory tool for early identification of high-risk infants, pending external validation, calibration assessment in independent cohorts, and long-term outcome evaluation.

Further multicenter studies are warranted to validate these findings and to explore the underlying mechanisms.

## Data Availability

The original contributions presented in the study are included in the article/Supplementary Material, further inquiries can be directed to the corresponding author/s.
